# Perceived barriers and facilitators to preventing hospital‐acquired pressure injury in paediatrics: A qualitative analysis

**DOI:** 10.1111/jan.16002

**Published:** 2023-11-30

**Authors:** Tanesha A. Dimanopoulos, Wendy Chaboyer, Karin Plummer, Sharon Mickan, Amanda J. Ullman, Jill Campbell, Bronwyn R. Griffin

**Affiliations:** ^1^ NHMRC Centre of Research Excellence in Wiser Wound Care, Menzies Health Institute Queensland Griffith University Nathan Queensland Australia; ^2^ Children's Health Queensland Hospital and Health Service South Brisbane Queensland Australia; ^3^ School of Nursing and Midwifery Griffith University Nathan Queensland Australia; ^4^ Faculty of Health Sciences & Medicine Bond University Robina Queensland Australia; ^5^ School of Nursing, Midwifery and Social Work University of Queensland Saint Lucia Queensland Australia

**Keywords:** adverse event, hospital‐acquired, hospital‐acquired complication, multidisciplinary, nursing, Paediatric, pressure injury, tertiary hospital, wound care

## Abstract

**Aim:**

This qualitative study aimed to identify nurses' and allied health professionals' perceptions and experiences of providing hospital‐acquired pressure injury (HAPI) prevention in a paediatric tertiary hospital in Australia, as well as understand the perceived barriers and facilitators to preventing HAPI.

**Design:**

A qualitative, exploratory study of hospital professionals was undertaken using semi‐structured interviews between February 2022 and January 2023.

**Methods:**

Two frameworks, the Capability, Opportunity and Motivation Model of Behaviour (COM‐B) and the Theoretical Domains Framework (TDF), were used to give both theoretical and pragmatic guidance. Participants included 19 nursing and allied health professionals and data analysis was informed by the framework approach.

**Results:**

Analysis revealed nine core themes regarding professionals' beliefs about the barriers and facilitators to HAPI prevention practices across seven TDF domains. Themes included HAPI prevention skills and education, family‐centred care, automated feedback and prompts, allocation and access to equipment, everybody's responsibility, prioritizing patients and clinical demands, organizational expectations and support, integrating theory and reality in practice and emotional influence.

**Conclusion:**

These findings provide valuable insights into the barriers and facilitators that impact paediatric HAPI prevention and can help identify and implement strategies to enhance evidence‐based prevention care and prevent HAPI in paediatric settings.

**Impact:**

Overcoming barriers through evidence‐based interventions is essential to reduce HAPI cases, improve patient outcomes, and cut healthcare costs. The findings have practical implications, informing policy and practice for improved preventive measures, education, and staffing in paediatric care, ultimately benefiting patient well‐being and reducing HAPIs.

**Patient or Public Contribution:**

No patient or public contribution. The focus of the study is on healthcare professionals and their perspectives and experiences in preventing HAPIs in paediatric patients. Therefore, the involvement of patients or the public was not deemed necessary for achieving the specific research objectives.

## 1 BACKGROUND

Hospital‐acquired pressure injuries (HAPIs) are mostly regarded as a condition of adult patients but are also prevalent among children (Delmore et al., 2019). HAPI incidence in paediatrics has been reported as varying between 3.7% and 27% (Triantafyllou et al., 2021; Zhang et al., 2022). While the sequelae vary with severity, these injuries can have significant consequences, including prolonged hospital stays (Hauck et al., 2017), emotional challenges, pain and discomfort and compromised quality of life (Gorecki et al., 2011; Kim et al., 2019; Kottner et al., 2019). HAPIs also result in an increased healthcare delivery burden, with the estimated total cost of HAPI treatment in Australia for 2020–2021 being $9.11 billion per annum (Nghiem et al., 2022). Padula and Delarmente (2019) reported the average costs related to HAPI in the United States to be $26.8 billion, of which the majority (59%) was attributed to the more serious Stage 3 and 4 injuries. These findings underscore the importance of focusing on prevention and early intervention to achieve significant cost reduction (Padula & Delarmente, 2019). Extrinsic risk factors for HAPIs include the duration and amount of pressure, the presence of moisture and abnormal positions as well as intrinsic factors such as anaemia, oedema, poor nutrition and immobility (Baharestani & Ratliff, 2007; Mallick et al., 2023).

Along with screens like psychological, nutrition and pain screens, HAPI risk assessments are integral in guiding HAPI prevention and early intervention (Padula & Delarmente, 2019; Triantafyllou et al., 2021) and are the most effective way to improve patient outcomes and reduce hospital costs (Boylan, 2020). According to Kottner et al. (2013), at least 12 pressure injury risk assessment tools have been developed for paediatric patients (Kottner et al., 2013). The Glamorgan Risk Assessment Scale and the Braden Q Scale are the most commonly used pressure injury risk assessment scales in the paediatric healthcare field. Both risk assessment tools have been validated for sensitivity and specificity (Noonan et al., 2011; Vocci et al., 2023; Willock et al., 2007) and appear to be effective in neonatal, paediatric intensive care and general children's wards (Willock et al., 2007). Additionally, the Neonatal Skin Risk Assessment Scale (NSRAS) has been tested for sensitivity and specificity in the neonatal population, but questions have been raised about its validity, possibly due to a relatively small sample size (Broom et al., 2017; Huffines & Logsdon, 1997). Paediatric patients and neonates have similar risk factors as adult patients for pressure injury, with some additional considerations due to their anatomical and physiological differences (Freundlich, 2017). Paediatric patients are also particularly vulnerable to developing HAPIs in the hospital setting due to other unique risk factors such as skin immaturity and fragility, medical devices, and long hospitalisations (August et al., 2021; August et al., 2023).

As HAPIs are considered an indicator of quality care, evidence‐based international clinical guidelines provide a clear structure for prevention and advocate for nursing, allied health and medical professionals to be involved in their prevention (Australian Commission on Safety and Quality in Health Care, 2012; European Pressure Ulcer Advisory Panel et al., 2019; National Clinical Guideline Centre (UK), 2014). However, many international clinical guidelines are primarily intended for adult patients, and the majority of HAPI preventative care for paediatric patients has been extrapolated from practices developed for adults. These clinical guidelines create opportunities for potential harm by failing to consider the small but important number of paediatric risk factors (Baharestani & Ratliff, 2007; Tawhara & Forster, 2021).

While HAPI prevention is generally nurse‐led (Ebi et al., 2019; Usher et al., 2018), researchers have shown that allied health professionals are competent and willing to contribute to HAPI prevention (Macens et al., 2011; Worsley et al., 2017). Despite this potential, they are not frequently involved in HAPI prevention, and their role often remains uncertain in practical settings, representing an untapped and valuable resource (Macens et al., 2011; Worsley et al., 2017). Researchers have previously reported that HAPI prevention is an important part of standard nursing care, and nurses generally have a positive attitude towards HAPI prevention, education and knowledge (Coyer et al., 2019; Strand & Lindgren, 2010). Adequate staffing, knowledge and staff cohesion, as well as an organizational commitment to monitoring care delivery, can contribute to HAPI prevention. However, high patient acuity and competing work demands make prevention strategies difficult to implement and a significant organizational challenge (Coyer et al., 2019; Strand & Lindgren, 2010). Educating both nursing and allied health professionals about risk factors, management, and prevention strategies for HAPIs, as well as utilizing validated risk assessment tools and developing best practice guidelines, is essential for the prevention of HAPIs.

While HAPI prevention bundles and nursing interventions have been proven effective in reducing HAPIs in both adults and paediatrics (Gaspar et al., 2019; Tawhara & Forster, 2021), their uptake remains inconsistent (Chaboyer et al., 2017; Martinez‐Garduno et al., 2019). This has significant ramifications for clinical practice and the allocation of resources, particularly considering that a large portion of HAPI may be avoidable (Rowe et al., 2018). More research is needed regarding prevention strategies specifically to reduce HAPIs in paediatric settings and to better understand what aids and hinders HAPI prevention, including factors that influence clinical behaviour. Planned and facilitated implementation of evidence and guidelines has been found to improve the uptake and use of evidence (Bunce et al., 2020; Roberts et al., 2017), while behaviour change is more effective if based on evidence‐based principles (Cane et al., 2012). The aim of this study was to explore nursing and allied health professionals' perceptions and experiences towards providing HAPI prevention in a paediatric tertiary hospital in Australia, as well as to understand the perceived barriers and facilitators in preventing HAPI.

## 2 METHOD

### 2.1 Study design

A qualitative, exploratory study of hospital professionals was undertaken using semi‐structured interviews. Qualitative research designs have made significant contributions to nursing and healthcare practices and policies, providing an excellent method to address issues of clinical significance like HAPI prevention (Doyle et al., 2020).

### 2.2 Ethical considerations

Approval for the conduct of this study was obtained from the Children's Health Queensland Hospital and Health Service Human Research Ethics Committee (HREC/21/QCHQ/80519) on the 4 November 2021, and Griffith University (2021/919) on the 8th of February 2022. This study was reported according to the consolidated criteria for reporting qualitative studies (COREQ) (Supplementary File 1).

### 2.3 Conceptual framework

**FIGURE 1:**
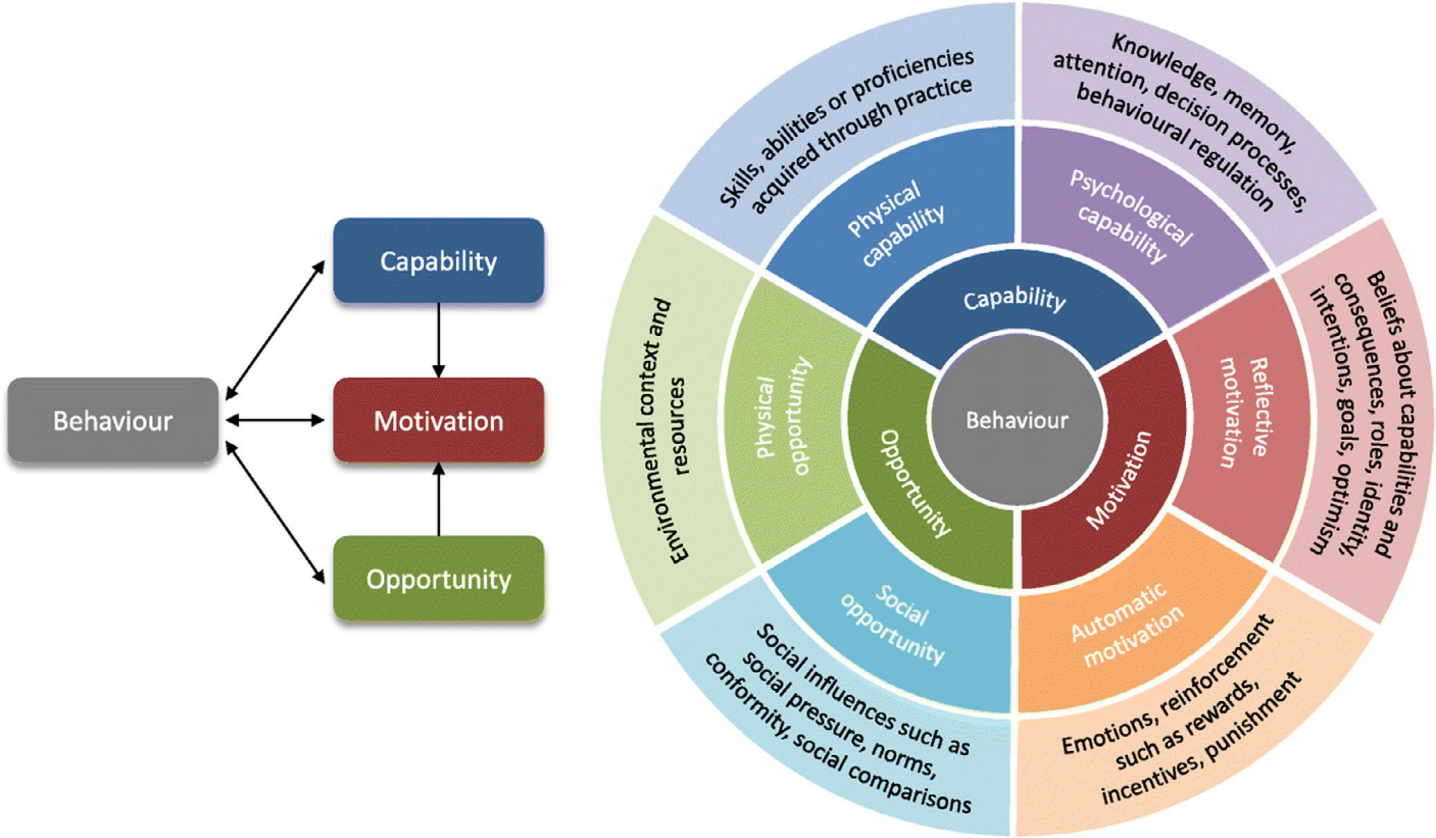
COM‐B domains and TDF domains. Reproduced with permission from McDonagh et al. (2018).

To provide both theoretical and pragmatic guidance for this study, we used two complementary frameworks: the Capability, Opportunity and Motivation Model of Behaviour (COM‐B) (Cane et al., 2012) and the Theoretical Domains Framework (TDF) (Michie et al., 2005) (Figure 1). The COM‐B model is situated within the Behaviour Change Framework alongside intervention functions and policy categories. The model is a simple but flexible tool that aims to identify deficits in a target behaviour, in our case, paediatric HAPI prevention and what needs to shift to achieve the desired change (Michie et al., 2014). The COM‐B is heavily aligned with the TDF, an integrated theoretical framework that has been used across a wide range of healthcare settings (Cane et al., 2012; Michie et al., 2014). The TDF aims to identify influences on many clinical behaviours related to the implementation of evidence‐based recommendations and facilitate behaviour change interventions in different healthcare contexts (Lavallée et al., 2018; Wu et al., 2023). These two frameworks have been used to identify barriers and facilitators to behaviour and design interventions in many contexts, including HAPI prevention in the adult population. However, there is a paucity of research into HAPI prevention in paediatric hospitals and it has yet to be applied in this setting.

### 2.4 Study setting and participants

This study was conducted at an Australian tertiary paediatric hospital between February 2022 and January 2023. Purposive sampling was initially used to recruit participants from orthopaedic, neuroscience, rehabilitation and intensive care units (Etikan et al., 2016). These units were selected as it has been consistently demonstrated that patients are at high risk of developing HAPIs in these units (European Pressure Ulcer Advisory Panel et al., 2019). Nursing and allied health directors and managers from these units were approached for permission to contact health professionals with varying expertise and experience in pressure injury prevention. This included frontline and managerial nursing professionals, dietitians, occupational therapists, and physiotherapists. The purpose of the study was introduced to health professionals via email. Additionally, snowball sampling was utilized (Etikan et al., 2016) and health professionals were encouraged to forward the study information to their interested colleagues from other units. Initial invitation emails were sent to 32 professionals; the main reason for non‐participation was scheduling and time issues.

### 2.5 Data collection

The semi‐structured interview guide (Supplementary File 2) was developed based on the research objectives and informed by the COM‐B model. A total of 14 audio‐recorded interviews were conducted by the first author between May and July 2022. A secondary female interviewer was also present at three interviews. Of these 14 interviews, two were focus groups with three participants, one was a focus group with two participants and the rest were individual (*n* = 11). The majority of interviews occurred face‐to‐face at the hospital, in private clinic rooms at the preference of the participant. One interview occurred over zoom due to COVID‐19 isolation restrictions. Potential participants were provided with a copy of the Participant Information and Consent Form prior to the interview and given the opportunity to peruse and ask questions. These consent forms were signed just before the commencement of the interview. Each interview ranged from 20 to 50 min in length and was deemed complete when all key points from the interview guide had been discussed and the participant had no additional questions or comments. Demographic information like gender, clinical role and unit was collected at the time of the interview.

The sample size was initially estimated at 10–15 interviews based on the recommendation by Francis et al. (2010) and previous health research using the TDF and COM‐B models (Francis et al., 2010; Hennink & Kaiser, 2022; Pearse et al., 2021). However, the final sample size was determined through data sufficiency, not data saturation (Vasileiou et al., 2018). Data sufficiency focuses on having an adequate amount and variety of data to address research objectives and provide comprehensive insights. Conversely, data saturation is the point at which new data collection is no longer revealing substantially new information (Hennink & Kaiser, 2022; LaDonna et al., 2021; Vasileiou et al., 2018). We chose sufficiency over saturation to align with our research goals of deepening understanding within the specific area of interest without the need to refine theory. This approach prioritizes credibility and coherence over artificially claiming saturation (Hennink & Kaiser, 2022). In the present study, data collection and analysis occurred concurrently. At interview 14, researchers deemed that the data set was sufficiently rich to not only identify themes and answer the research question but also understand them and account for differing perspectives (Hennink & Kaiser, 2022; LaDonna et al., 2021; Vasileiou et al., 2018).

### 2.6 Rigour and reflexivity

Each interview was conducted by a trained, female research assistant with a post‐graduate qualification in health, and skills in qualitative research. A secondary female interviewer, a trained emergency paediatric nurse and experienced qualitative researcher, oversaw this process and was also present at three interviews. The researchers had no authority or reporting relationship with the participants prior to the interviews, allowing for honest and open interview discussions.

To enhance the credibility of the findings, member checks were employed where participants were given the opportunity to review and edit their responses following the interview (Tobin & Begley, 2004). The inclusion of focus groups as an additional method of data collection enabled qualitative method triangulation and enhanced rigour. Method triangulation can increase the trustworthiness of findings and in our case, provide a deeper understanding of both individual and contextual circumstances (Lambert & Loiselle, 2008). A diversity of sources was also employed as our sample included multidisciplinary professionals, incorporating the perspectives of various health professionals with differing backgrounds and roles (Patton, 1999).

A reflexive journal was kept by the first author in the form of an electronic diary used to track interview scheduling and document written reflections, memos and fieldwork notes throughout the research process (Olmos‐Vega et al., 2023). The majority of the authors had extensive experience and knowledge in conducting research into wound care and qualitative research. In order to recognize the presence of potential bias due to topic interest and previous knowledge of paediatric HAPIs, the authors continuously reflected and participated in collaborative reflexivity during the interview, analysis and write‐up process to ensure a true reflection of the data (Olmos‐Vega et al., 2023).

### 2.7 Data analysis

The interviews were audio‐recorded and transcribed using a hybrid approach. Microsoft Teams was used for recording and initial transcription. Transcripts were then reviewed and cleaned by a member of the research team to ensure accuracy of transcription. Data analysis was informed by the framework approach (Gale et al., 2013; Ritchie & Spencer, 2002) to systematically identify new emerging themes while utilizing the theoretically driven domains of the TDF and COM‐B models (Cane et al., 2012; Michie et al., 2014).

The first step of the analysis involved the first author and a senior researcher with experience in qualitative data analysis reviewing the transcripts and recordings and immersing themselves in the data. The two researchers coded the first three transcripts independently to compare coding consistency. To develop a thematic framework, codes and early themes were identified inductively from professionals own reported experiences regarding the contextual determinants of effective prevention of paediatric HAPI and deductively based on the constructs and domains of the TDF framework (Cane et al., 2012; Michie et al., 2014). A ‘working’ codebook and thematic framework were created in NVivo 10. This coding framework was applied to all remaining transcripts. This was a flexible and iterative process with further coding and meetings refining the final thematic framework. Researchers then indexed the transcripts by grouping together all codes that shared structures or consistencies. This created themes and sub‐themes, which were then mapped back to the TDF and summarized using the simpler format of COM‐B.

The mapping and grouping of codes to the TDF domains showed minor discrepancies, especially when codes were mapped to multiple domains. When disagreements arose, the wider research team, including a researcher with extensive experience using the TDF and COM‐B models was brought in for discussion and to reach an agreement. Data were then charted in framework matrices to summarize and compare professionals' experiences of providing effective HAPI prevention, organize the illustrative quotes and provide an overall interpretation of the findings.

## 3 RESULTS

### 3.1 Sample

A total of 19 health professionals took part in the study, including 12 registered nurses (4 Nurse Unit Managers, 7 Nursing Clinical Leaders and 1 Registered Nurse (RN)) and 7 allied health professionals (2 Dietitians, 2 Physiotherapists and 3 Occupational Therapists). Two participants were male. Participants came from various specialities and clinical contexts, including rehabilitation (*n* = 6), paediatric intensive care (*n* = 4), surgery (*n* = 2), operating room suite (*n* = 1), emergency department (*n* = 1), oncology and palliative care (*n* = 1), orthopaedics (*n* = 1), neurosurgery (*n* = 1) and neurosciences (*n* = 1). One participant was an allied health consultant who reported overseeing multiple clinical specialties.

### 3.2 Research findings

Analysis revealed nine themes regarding professionals' beliefs about the barriers and facilitators to HAPI prevention practices. These themes span seven domains of behaviour influencing the uptake of paediatric HAPI prevention and the three main components of the COM‐B model. Results are presented thematically under relevant COM‐B headings to organize the findings. Additionally, an overview of identified themes and links to the relevant COM‐B and TDF domains are presented in Table 1.

**TABLE 1:** Overview of analysis showing themes and sub‐themes mapped to COM‐B and TDF.

COM‐B construct	TDF domain	Themes	Sub‐themes – identified as barriers or facilitators
Capability	Skills and knowledge	1. HAPI prevention skills and education	Facilitator: Good understanding of hospital‐acquired pressure injury
			Barrier: Lack of confidence in staging
			Barrier: Importance of education and training, but a lack of focus on paediatrics
		2. Family‐centred care	Facilitator: Prevention education for families and recognizing importance of patient and family‐centred care
		3. Automated feedback and prompts	Facilitator: Importance of feedback and learning from each other
			Barrier: Electronic programs offer automated prompts and notifiers
Opportunity	Environmental context	4. Allocation and access to equipment	Barrier: Inequitable allocation and access to equipment
	Social influences	5. Everybody's responsibility’	Facilitator: Multi‐disciplinary team approach with good communication
		6. Prioritizing patients and clinical demands	Barrier: Prioritizing pressure injury prevention vs other nursing demands
			Barrier: Risk versus benefits of medical intervention
			Barrier: Generating alternatives for complex patients
Motivation	Social/professional role and identity	7. Organizational expectations and support	Facilitator: Support from stomal therapy team
			Barrier: Uncertainty of expectations and roles
	Intention	8. Integrating theory and reality in practice	Barrier: Lack of coherence in planned care due to a complex system
	Reinforcements	8. Integrating theory and reality in practice	Barrier: Sensitivity of risk assessment tools
			Barrier: No professional ramification or reward
	Emotion	9. Emotional influence	Facilitator: Salient experiences
			Barrier: Increased workload and time pressures

#### 3.2.1 Capability

##### HAPI prevention skills and education

The majority of professionals reported a good understanding of HAPIs and described skills such as regular skin checks and routine monitoring of medical devices as important for prevention. Health professionals recalled previous success in preventing HAPIs from worsening by identifying changes in skin integrity early and intervening. However, nurses also discussed their concerns over the accurate staging of paediatric HAPIs. They reported a lack of confidence and accuracy regarding staging, and this skill set was described as role‐dependent or based on the nurse's experience level, as explained by the following nurse:

You know, there are some of the nurses out there that are very good at doing it. And then there's other nurses who just that's not part of their care (P4).

Health professionals highlighted the importance of continuing education and training for HAPI prevention. There was a consensus that specialized HAPI education could be improved and the lack of mandatory education for all professionals was a barrier. Paediatric‐focused education was also a barrier, with health professionals relying on clinical experience and incidental learning instead of formal upskilling and education as evidenced in the following quote:

It's quite specialised so not really that I, cause I kind of just picked it up through clinical experience through liaising with stomal therapists, through reading articles I haven't really, I don't think I've attended anything specific … especially in relation to Paediatrics (P10).

##### Family‐centred care

Many health professionals spoke about the principles of family‐centred care and the importance of empowering patients and their families. Health professionals perceived that verbally educating and engaging patients and families in their care led to effective HAPI prevention through knowledge of risk factors that parents could ‘escalate to the bedside nurse’ (P17). Health professionals described a symbiotic relationship, with families as the ‘experts’ and their involvement as a valuable resource for successful HAPI prevention:

We do have a lot of children that have got chronic disabilities and their families are, in fact, the expert … And they know they know their child, they know how to position, how this works and we're utilising that all the time (P16).

##### Automated feedback and prompts

The occurrence of HAPI seemed infrequent in the current context, as many health professionals reflected on previous high occurrences and how rates have lowered. Information on HAPI occurrences was seen as important feedback and many health professionals spoke enthusiastically about the benefits of visibility and learning from each other. Feedback was seen as an educational opportunity for improving and changing practice as one health professional explained that once neglected factors were identified then ‘we can modify practice in terms of education to families and what we do’ (P14).

Across the interviews, professionals frequently mentioned the integrated electronic Medical Record system (IeMR) used by the hospital as ‘a comprehensive system in the way it flags with you’ (P15). The electronic system offers automated prompts on when to complete risk assessments and provides notifications when patients return high‐risk HAPI screens. The majority of health professionals found these prompts helpful as reminders to complete HAPI risk screens, however, the completion of the risk screens was mentioned by some professionals as automatic and ‘something that we kind of just do out of habit’ (P8). Additionally, some participants reported a fatigue with risk screens pop‐ups and their automated nature, resulting in them being ignored and therefore not integrated into care:

Yeah, there's a lot. I know that some of the pop ups I know I ignore them because I'm like yes, I know exactly which pop up that is, it won't make a change to me (P4).

#### 3.2.2 Opportunity

##### Allocation and access to equipment

Health professionals reported that alternating pressure mattresses, gel pads, cushions, pillows and Turn and Position systems were essential for preventing HAPI. Some professionals reported being well equipped with HAPI prevention equipment, while others described access to equipment as ‘tricky’ and ‘challenging’ leading to some departments becoming protective of equipment and unwilling to lend it out. One participant commented how ‘I'm not going up to PICU because they're so protective. So, then we get territorial with our equipment’ (P7). Patients leaving high‐acuity wards also presented challenges for professionals due to inequitable equipment provisions, making transitions of care opportunities for risk. This challenge was also illustrated for high‐risk patients with their own specialized equipment in the transition from community to hospital. Health professionals reflected on the expectation from patients and carers to supply the same standard of equipment in a hospital setting and their inability to meet this expectation:

Part of it is probably this belief that, well, you're a [sic] health service. You should have everything to access for my child while I'm there. Like when I go to stay at a hotel, I don't expect to take my own sheets (P19).

##### Everybody's responsibility

Professionals reported a close working relationship between nursing and allied health professionals with HAPI prevention seen as being nurse‐led but ‘everybody's responsibility’ (P8). The opportunity to collaborate as a multidisciplinary team was seen as a strength and the importance of good communication was ‘an enabler to make sure that we reduce the risk of pressure area development’ (*P18*). Professionals believed that fragmented written and verbal communication negatively influenced their ability to implement HAPI prevention. Compounding these barriers were inadequate documentation and sub‐optimal handovers, which hinder patient visibility as exemplified in the following quote:

We won't have any risk assessments or any knowledge of things that are happening other than it just being sort of verbally passed along the line like there's, we just, everything's done in umm, just little chunks with no communication at all (P7).

##### Prioritizing patient needs and clinical demands

Professionals described a system of prioritizing between completing quality HAPI prevention and other clinical duties and responsibilities, sometimes at the expense of patient care and compliance with best practice. One allied health professional mentioned an ‘efficiency, thoroughness trade‐off’ (P18) between completing mandatory risk assessments and taking professionals away from the bedside ‘which is stopping them doing something else that's really important, which creates a risk for some other area’ (P18). Amidst the discussion of barriers to HAPI prevention, the delicate balance between the risks and benefits of medical intervention was mentioned by multiple professionals. HAPIs were sometimes seen as an inevitable result of crucial medical devices; however, the benefits of these devices outweighed the risk of PI, enforcing the need for thorough HAPI prevention:

We do everything we can. So, we know that this is a risk. If we didn't have it, it would be more a risk, if we need, it's weighing up the risk benefit, right. So, we need it on because it's gonna keep our hips and pelvis in the right position. But there is a risk there. So, it's about checking the skin regularly (P14).

Almost all health professionals identified patient comorbidities like incontinence, malnourishment and neuromuscular disorders as obstacles to providing HAPI prevention. The anatomical and physiological differences between paediatric and adult patients were also discussed in most interviews. Most health professionals acknowledged these added complexities as recurrent and serious obstacles to providing HAPI prevention. Health professionals described adapting and critical thinking to generate alternatives for these patients, such as completing micro turns or repositions instead of full turns and modifying incorrectly sized equipment and devices:

It's adapting. And we've got, I've got a few kids who are essentially got neuropathic ulcers and everything you do is all about the adults [guidelines], so it's all about adapting (P14).

#### 3.2.3 Motivation

##### Organizational expectations and support

Most professionals saw HAPI prevention as an integral part of patient care, with the support provided by the stomal therapy team being crucial in mitigating risk. However, there was some uncertainty among professionals regarding the professional boundaries of their own and others' clinical practice. Professionals expressed frustration with colleagues' ‘not my job’ mindset and believed that more transparent policies and standards would help them understand expectations. Places like the Emergency Department and Operating Theatre Suites were seen as transient stopping points for patients, with limited responsibility and ownership over HAPI. One nurse mentioned that specific HAPI prevention strategies were sometimes missed as a result:

Well, I think it's mainly to do with pressure areas. I think there's a perception that if they're coming to theatre, you don't need to do a full handover… Only for a shorter time. But there's things that are missed because of that… We're often forgotten, yes very much often forgotten up here (P8).

Some professionals cited the Clinical Nurse Consultant (CNC) model as a crucial HAPI prevention factor, acting as an expert for support, oversight, and gatekeeper before the stomal therapy team. Traditionally, a CNC is a Registered Nurse with post‐registration nursing qualifications relevant to their speciality field. However, some professionals cited a different model of care, with less‐qualified professionals like undergraduate nursing students and Assistants in Nursing completing skin checks to increase HAPI prevention compliance and visibility.

##### Integrating theory and reality in practice

Professionals mentioned a lack of coherence in planned care and described a complex system where HAPI risks are identified and strategies are implemented during regular working hours, but not utilized after‐hours. For example, an allied health professional shared an incident where despite implementing all the HAPI prevention staff were aware of, a patient still developed a HAPI:

We thought we'd mitigated that with education to parent to say, look, if you got any problems, come on back. And the parent did that. But then it fell down. She did what she, what she could do, what she was required to do…. And it fell down because we didn't kind of use all of our resources here at the hospital because the person who looked at them didn't think about OT on call because that wasn't in their sphere of knowledge and awareness at the time. So, they just tried to problem solve around in what they thought they had (P19).

Many nurses highlighted that the Glamorgan Risk Assessment Scale lacked sensitivity for this population, and recommended pressure‐relieving equipment for too many patients. Professionals described the scale as inadequate as a paediatric risk assessment tool and described the organizational requirement to complete the screen as prescriptive with a lack of proper meaning regarding implementing HAPI prevention. Professionals highlighted the importance of using their clinical judgement in conjunction with risk assessments for effective HAPI prevention. Still, they felt the mandatory nature of the Glamorgan screening tool took away their clinical decision‐making and restricted their risk management abilities. Multiple professionals referred to the ‘tick a box’ risk assessments as adding extra work instead of guiding HAPI prevention strategies contributing to best practice. Additionally, there was no professional ramification or reward for following the Glamorgan Screen, which some professionals believed affected behaviour:

So, you know, there's kind of the real world and then there's the tick a box stuff that we have and we're very tick a box here. We want to make sure you tick the box in ieMR. We want to make sure that you've done X, Y & Z. But does that translate to best practice? You know, no (P7).

##### Emotional influence

Professionals reported being highly motivated to deliver high‐quality HAPI prevention. However, instead of motivation from the organization, nurses referred to previous, salient experiences as heavily influencing their HAPI practices. Many health professionals recalled serious HAPIs they had treated throughout their careers, the harm they had caused, and their desire to avoid injuries. One nurse stated they had seen ‘the end result and our preference is to not cause pressure injuries. We've had those kids in the past. We don't want anymore’ (P16).

Although health professionals voiced concerns about the negative impacts of HAPI and seemed motivated to deliver high‐quality HAPI prevention, increased workload and time pressures were seen as barriers to the behaviour. Health professionals described being tired with staff shortages and high turnover making it challenging to complete the required HAPI prevention; this included log‐rolling larger patients, which required multiple health professionals to complete safely. Although they had not noticed a tangible increase in HAPI, some health professionals mentioned absorbing additional work which could potentially compromise their ability to effectively prevent HAPI:

And I think that's in general a main impact on the ward, but it hasn't, it hasn't resulted in increased pressure injuries or anything like that I think they've just absorbed it into their workload, which everyone's, at the moment, is quite heavy and quite tired, but we've absorbed that I would say, yeah, all that extra stuff (P3).

## 4 DISCUSSION

HAPIs are a serious concern for healthcare providers due to their frequency and their potential to cause significant harm (Gorecki et al., 2011; Hauck et al., 2017; Kim et al., 2019; Kottner et al., 2019). This exploration of contextual factors using the COM‐B and TDF frameworks highlights the complexity and challenges of paediatric HAPI prevention in an Australian hospital setting. To the best of our knowledge, this is the first Australian study to report qualitative data from hospital professionals regarding barriers and facilitators of clinical practice in the prevention of paediatric HAPI. This study identified nine themes across seven theoretical domains of behaviour that influence the uptake of paediatric HAPI prevention and the three main components of the COM‐B model.

The capability and capacity of professionals to identify and prevent HAPIs is crucial. We found a key facilitator to prevention was a good awareness of the knowledge and skills required for pressure injury prevention, which is consistent with prior research conducted in adult intensive care unit (ICU) populations (Coyer et al., 2019; Li et al., 2022; Strand & Lindgren, 2010). We identified that professionals had little to no opportunity to participate in continuing education activities, especially paediatric‐specific training. This may explain the reported lack of confidence in staging identified and highlights the need for staff education and training (Cummins et al., 2019). Prior studies have emphasized the necessity of population‐specific HAPI education, particularly in neonates, which requires a highly specialized workforce due to diverse HAPI risks (August et al., 2020; Liversedge et al., 2018).

Our findings emphasize the need to prioritize patient‐centred care, including involving patients and their families in preventing and managing HAPIs. The family‐centred care nurses provide is an essential aspect of HAPI prevention, especially in busy hospitals where health professionals have multiple priorities. One of the most significant findings of this study was the need for more awareness among healthcare professionals about the differences between monitoring the risk of paediatric and adult HAPIs, with professionals relying on clinical experience and incidental learning instead of formal upskilling and education. A study by August et al. (2020) similarly identified that clinician experiences have a stronger influence on the identification and treatment of neonatal HAPI compared to formal education (August et al., 2020). These findings highlight the need for healthcare organizations to implement comprehensive and tailored training programs that foster a family‐centred approach and address the unique challenges posed by paediatric HAPIs. This training will enable professionals to move away from relying on experiential learning and enable evidence‐based identification and treatment of HAPIs in this vulnerable population.

Environmental context and social influence barriers, including inadequate staffing, the allocation of equipment and the presence of devices, highlight the need for adequate staffing levels, equipment and resources to support prevention practices. This builds upon prior research examining safe and effective staffing levels in healthcare; however, despite extensive evidence demonstrating strong associations between sub‐optimal levels of nurse staffing and increased rates of adverse events, including mortality, causal links have not been established (Griffiths et al., 2016; Twigg et al., 2019; Wynne et al., 2021). Motivating factors, such as a multidisciplinary team approach and the importance of communication, were identified as facilitators. This is not a new finding, with Wu et al. (2023) and Li et al. (2022) reporting that social support provided by multi‐disciplinary teams with clearly defined roles is perceived as a facilitator in the uptake of HAPI prevention in adults. This highlights the need for a culture of safety that values prevention practices, leadership support and prioritization of prevention practices to address these barriers and promote safety (Li et al., 2022; Wu et al., 2023).

The present study produced some unique and important findings. Professionals reported that complex, acute patients offered challenges in providing HAPI prevention and mentioned offering innovation and generating alternatives. Health professionals acknowledge major gaps in the delivery of HAPI prevention, yet they continue to ‘work around’ the barriers to provide optimum care and do what they can for best patient outcomes. Health professional highlighted insufficient risk assessment sensitivity and a lack of accountability in the completion of assessments as key barriers to effective prevention of HAPI. Identifying the patient's level of risk is essential for effective prevention of PI, as it informs prevention strategies, and thereby risk reduction (Boylan, 2020; Triantafyllou et al., 2021). This lack of accountability was also reflected in a study by Strand and Lindgren (2010), who found that a reported lack of routines, accountability and social pressure for HAPI risk assessments made them less likely to be performed (Strand & Lindgren, 2010). This present study has found that health professionals perceived the tool to be void of value and reported it as insufficient to guide clinical care. The perception of the low sensitivity of the tool fosters obligatory completion for documentation purposes rather than providing meaningful support for clinical decision‐making.

However, the Glamorgan scale stands as a strong option for assessing HAPI risk in paediatrics, owing to its well‐established validation (Noonan et al., 2011; Vocci et al., 2023; Willock et al., 2007). In a study examining the predictive diagnostic value of the Waterlow, Braden Q and Glamorgan scales, the Braden Q and Waterlow scales had better specificities than the Glamorgan scale (Luo et al., 2021). It was also illustrated that the Waterlow scale may present the best overall accuracy in a paediatric ICU setting, whereas the Braden Q and Glamorgan scales might have greater accuracy in a general paediatric ward context (Luo et al., 2021). There is also some evidence that the Glamorgan scale has low inter‐rater reliability in low acuity contexts, however, the authors suggest this may be a result of the low overall HAPI risk in this setting and did not recommend the tool for daily use (Kottner et al., 2014). The reliability, accuracy and specificity of the Glamorgan scale, in combination with our finding that professionals view the Glamorgan scale as an inadequate paediatric risk assessment tool, especially in the ICU, questions the use of the Glamorgan as a blanket tool for all patients regardless of acuity within our hospital context.

### 4.1 Strengths and limitations

This study has several strengths. Our findings support and extend those of previous studies exploring the barriers and facilitators of HAPI prevention in different contexts, while also offering novel and unique findings. Furthermore, the use of behaviour change frameworks such as the COM‐B and TDF provides a more detailed and theory‐based understanding of the context and behaviours involved in the prevention of paediatric HAPI. Another important strength of this study was the development of a thorough understanding of the contextual factors influencing clinical practices in this specific local area. Therefore, the effectiveness of research translation and the facilitation of the identification of targeted strategies depend critically on an understanding of the local context. Our inclusion of both nursing and allied health professionals captured multiple viewpoints and provided a richness in data missing from many other HAPI‐focussed research. A potential limitation is the exclusion of medical staff and family members perspectives, however, this enabled the current study to create a more detailed understanding of the perceived barriers and enablers of paediatric HAPI prevention reported by the health professionals who are primarily responsible for pressure injury prevention at the bedside (Macens et al., 2011; Worsley et al., 2017).

This study is limited in generalisability by the nature of its qualitative design in a single, tertiary paediatric setting. We also recognize that some professionals have a particular interest in HAPI, and it is possible their views may not adequately represent those of the wider hospital. However, given the diverse characteristics of the professionals interviewed, the findings of the present study are likely to represent the part of the hospital workforce with the most HAPI experience. Ultimately, caution should be taken with the interpretation of the findings, but despite these limitations, this is a useful first step in understanding professionals' perspectives on the issues associated with providing HAPI prevention in a tertiary paediatric setting.

### 4.2 Implications for policy and practice

Without a comprehensive understanding of paediatric HAPI preventive barriers and facilitators, healthcare providers risk using ineffective strategies, resulting in unnecessary injury, increased healthcare expenses, and diminished quality of life for paediatric patients. This study identified barriers to pressure injury prevention and implies that effective paediatric HAPI prevention will require a more complex intervention using concerted behavioural planning that is multifaceted and multidisciplinary. It is crucial for healthcare organizations to prioritize and invest in resources that address these barriers to improve patient outcomes and reduce the burden of preventable HAPIs. By addressing these barriers and implementing evidence‐based interventions, healthcare providers can work towards reducing the prevalence of HAPIs in paediatric patients and ultimately improving patient outcomes.

### 4.3 Recommendations for further research

The accuracy and sensitivity of the Glamorgan tool are worthy of attention, as there is little current research on whether RNs perceive the Glamorgan as a barrier to HAPI prevention and care. Valuable additional research could focus on the identification of strategies and future interventions likely to improve the implementation of paediatric HAPI prevention among hospital professionals. The Behaviour Change Wheel consists of three layers: the COM‐B model, intervention functions, and policy categories. Further mapping of the COM‐B domains to the Behaviour Change Wheel will allow for the identification of intervention options, and the selection of strategies to improve effective HAPI prevention in clinical practice (Michie et al., 2014). Exploring the barriers and facilitators to HAPI prevention in paediatrics will inform strategies to enhance the implementation of HAPI prevention within our study setting and beyond.

## 5 CONCLUSION

These findings provide valuable insights into the barriers and facilitators that impact the prevention of HAPI in the paediatric population, providing crucial insights into an area of research that has been relatively underexplored, especially in the paediatric context. The analysis contributes to the broader body of knowledge on HAPI prevention and emphasizes the need for a comprehensive approach involving education, adequate staffing, leadership support, and reinforcement to promote prevention practices. Conceptualizing the findings more widely within the context of the COM‐B model and TDF framework offers a more detailed analysis of the barriers and facilitators, which can help to better target future pressure injury prevention strategies. This study's significance lies in its potential to inform targeted strategies, interventions, and policy changes that can enhance HAPI prevention in paediatric healthcare, ultimately reducing the burden of preventable injuries and improving overall patient care.

## AUTHOR CONTRIBUTIONS

Wendy Chaboyer, Amanda J. Ullman, Jill Campbell and Bronwyn R. Griffin contributed to the development and design of the study. Bronwyn R. Griffin acquired research funding. Tanesha A. Dimanopoulos, Wendy Chaboyer, Sharon Mickan, Amanda J. Ullman, Jill Campbell and Bronwyn R. Griffin were involved in data collection and analysis. Tanesha A. Dimanopoulos prepared the manuscript. All authors provided review and edits of the draft manuscript and approved the final manuscript.

## FUNDING INFORMATION

BRG, JC, WC and AU were funded by NHMRC Wiser Wounds CRE (APP 1196436), and TAD was funded by the Griffith University 2021 New Researcher Grant scheme.

## CONFLICT OF INTEREST STATEMENT

No conflicts of interest to declare.

## PEER REVIEW

The peer review history for this article is available at https://www.webofscience.com/api/gateway/wos/peer‐review/10.1111/jan.16002.

## ETHICS STATEMENT

Approval for the conduct of this study was obtained from the Children's Health Queensland Hospital and Health Service Human Research Ethics Committee (HREC/21/QCHQ/80519) and Griffith University (2021/919).

## Supporting information


**Supplementary file 1:** COREQ (COnsolidated criteria for REporting Qualitative research) Checklist.

**Supplementary File 2.** Semi‐structured interview guide.

## Data Availability

The data that support the findings of this study are available from the corresponding author upon reasonable request.
