# Supplementary material for: Perceived barriers and facilitators to preventing hospital‐acquired pressure injury in paediatrics: A qualitative analysis
Source: J Adv Nurs. 2023 Nov 30;81(11):7103–15. doi: 10.1111/jan.16002 (PMC12535351; doi:10.1111/jan.16002)
Supplement: Supplementary file 1 — COREQ (COnsolidated criteria for REporting Qualitative research) Checklist. [file JAN-81-7103-s002.docx]

| **Item** | **Guide questions/description** | **Page number/comments** |
| --- | --- | --- |
| **Domain 1: research team and reflexivity** | | |
| Personal characteristics | | |
| 1. Interviewer/facilitator | Which author/s conducted the interview or focus group? | 9: TAD and BRG. |
| 2. Credentials | What were the researcher’s credentials? e.g. PhD, MD | 9: TAD - Gdip (Public Health)  BRG – PhD. |
| 3. Occupation | What was their occupation at the time of the study? | 9: TAD - Research Assistant  BRG - Associate Professor and Principal Investigator. |
| 4. Gender | Was the researcher male or female? | 9: Interviewers were both female. |
| 5. Experience and training | What experience or training did the researcher have? | 9: Both interviewers had experience with qualitative methods including facilitating focus groups and interviews. |
| Relationship with participants | | |
| 6. Relationship established | Was a relationship established prior to study commencement? | 9: The researchers obtained informed consent from participants without prior contact or an existing professional relationship. |
| 7. Participant knowledge of the interviewer | What did the participants know about the researcher? e.g. personal goals, reasons for doing the research | 9: The purpose of the study was introduced to health professionals via email. All participants knew that the interview was for research purposes. |
| 8. Interviewer characteristics | What characteristics were reported about the interviewer/facilitator? e.g. bias, assumptions, reasons and interests in the research topic | 9: The interviewers both had experience and knowledge in conducting research into wound care and qualitative research. |
| **Domain 2: study design** | | |
| Theoretical framework | | |
| 9. Methodological orientation and theory | What methodological orientation was stated to underpin the study? e.g. grounded theory, discourse analysis, ethnography, phenomenology, content analysis | 6: The research team utilised framework analysis, analysing the data inductively without predetermined ideas. The COM-B and TDF frameworks were also used to guide understanding. |
| Participant selection | | |
| 10. Sampling | How were participants selected? e.g. purposive, convenience, consecutive, snowball | 7: Both purposive and convenience sampling were used. |
| 11. Method of approach | How were participants approached? e.g. face to face, telephone, mail, e-mail | 7: Via email. |
| 12.Sample size | How many participants were in the study? | 11: 19 health professionals |
| 13.Non-participation | How many people refused to participate or dropped out? Reasons? | 7: Invitation emails were sent to 32 health professionals, the main reason for non-participation was scheduling and time issues. No participants dropped out. |
| Setting | | |
| 14. Setting of data collection | Where was the data collected? e.g. home, clinic, workplace | 8: Face to face at the hospital, one interview occurred over zoom. |
| 15. Presence of non-participants | Was anyone else present besides the participants and researchers? | No other individuals were present in interviews |
| 16. Description of sample | What are the important characteristics of the sample? e.g. demographic data, date | 11: The important characteristics of the sample, like gender and role were reported. |
| Data collection | | |
| 17. Interview guide | Were questions, prompts, guides provided by the authors? Was it pilot tested? | 7: A semi-structured interview guide based on the research objectives and informed by the COM-B model was developed by the researchers. The guide was not pilot tested. |
| 18. Repeat interviews | Were repeat interviews carried out? If yes, how many? | Repeat interviews were not carried out in this study, and have therefore not been reported |
| 19. Audio/visual recording | Did the research use audio or visual recording to collect the data? | 7: All interviews were audio recorded with permission of participants. |
| 20. Field notes | Were field notes made during and/or after the interview or focus group? | 9: Researchers made field notes during the interviews and focus groups. These field notes were used to assist in the analysis of the transcribed audio recordings. |
| 21. Duration | What was the duration of the interviews or focus group? | 8: Interviews ranged from 20-50 minutes. |
| 22. Data saturation | Was data saturation discussed? | 8: Data saturation/sufficiency was discussed in the methods section. |
| 23. Transcripts returned | Were transcripts returned to participants for comment and/or correction? | 9: Participants had the option to review their transcripts, but none chose to do so. |
| **Domain 3: analysis and findings** | | |
| Data analysis | | |
| 24. Number of data coders | How many data coders coded the data? | 10: Two researchers coded the first three transcripts independently to compare coding consistency, the rest were then coded by one coder. |
| 25. Description of the coding tree | Did authors provide a description of the coding tree? | 10: There is no description of the coding tree. |
| 26. Derivation of themes | Were themes identified in advance or derived from the data? | 10: Themes were derived from the data in an inductive manner during analysis. |
| 27. Software | What software, if applicable, was used to manage the data? | 10: Researchers used NVivo during the coding of all interviews. |
| 28. Participant checking | Did participants provide feedback on the findings? | No participants provided feedback on the findings. |
| Reporting | | |
| 29. Quotations presented | Were participant quotations presented to illustrate the themes/findings? Was each quotation identified? e.g. participant number | 11-18: Key findings of this study were supported with selected quotations in-text. Each quotation is identified by a patient number. |
| 30. Data and findings consistent | Was there consistency between the data presented and the findings? | 11-18: All findings were derived from the data therefore there was consistency between the data presented and the findings. |
| 31. Clarity of major themes | Were major themes clearly presented in the findings? | 11-18: Results are presented thematically under relevant COM-B headings to organise the findings. |
| 32. Clarity of minor themes | Is there a description of diverse cases or discussion of minor themes? | 11-18: Minor themes with opposing opinions within and between groups are described in the results section. |
