# Supplementary material for: Perceived barriers and facilitators to preventing hospital‐acquired pressure injury in paediatrics: A qualitative analysis
Source: J Adv Nurs. 2023 Nov 30;81(11):7103–15. doi: 10.1111/jan.16002 (PMC12535351; doi:10.1111/jan.16002)
Supplement: Supplementary file 2 — Semi‐structured interview guide. [file JAN-81-7103-s001.docx]

Supplementary File 2. Semi-structured interview guide.

| **Introduction:** We know that pressure injuries can have significant complications and impact the quality of a child’s life. The importance of early and targeted prevention to avoid the development of serious pressure injuries that may impact on a child’s quality of life and psychosocial wellbeing is well understood. However, there are gaps in the current understanding of Australian clinician’s perspectives on helpful and unhelpful strategies for pressure injury prevention in these children. We are aiming to improve this gap.  Your knowledge of paediatric care including pressure injury prevention, and the current systems in this setting will help us improve this care in the future. Thank you for sharing your expertise with us today.  The interview will last approximately 30minutes. We ask that you do not reveal any patient or staff names, birthdates or use any other identifying information when answering questions |
| --- |
| 1. From your perspective what are some of the things that might facilitate the use and uptake of pressure injury prevention strategies? (Probes: evidence base, guidelines, ease of implementation etc ) |
| 2. From your perspective what are some of the things that you think are barriers to the use and uptake of pressure injury prevention strategies? (Probes: time, workload, competing priorities, knowledge, clinical capability, access to equipment) |
| 3. What are some of your ideas about how we could improve pressure injury prevention (Probes: supporting the benefits to patients amongst peers, education campaign, champions) |
| 4. Is there anything else you wish to share about the prevention of pressure injuries we have not discussed? |
